# A Real-Time Automated Patient Screening System for Clinical Trials Eligibility in an Emergency Department: Design and Evaluation

**DOI:** 10.2196/14185

**Published:** 2019-07-24

**Authors:** Yizhao Ni, Monica Bermudez, Stephanie Kennebeck, Stacey Liddy-Hicks, Judith Dexheimer

**Affiliations:** 1 Cincinnati Children's Hospital Medical Center Cincinnati, OH United States

**Keywords:** automated patient screening, system integration, natural language processing, time and motion studies, system usability evaluation

## Abstract

**Background:**

One critical hurdle for clinical trial recruitment is the lack of an efficient method for identifying subjects who meet the eligibility criteria. Given the large volume of data documented in electronic health records (EHRs), it is labor-intensive for the staff to screen relevant information, particularly within the time frame needed. To facilitate subject identification, we developed a natural language processing (NLP) and machine learning–based system, Automated Clinical Trial Eligibility Screener (ACTES), which analyzes structured data and unstructured narratives automatically to determine patients’ suitability for clinical trial enrollment. In this study, we integrated the ACTES into clinical practice to support real-time patient screening.

**Objective:**

This study aimed to evaluate ACTES’s impact on the institutional workflow, prospectively and comprehensively. We hypothesized that compared with the manual screening process, using EHR-based automated screening would improve efficiency of patient identification, streamline patient recruitment workflow, and increase enrollment in clinical trials.

**Methods:**

The ACTES was fully integrated into the clinical research coordinators’ (CRC) workflow in the pediatric emergency department (ED) at Cincinnati Children’s Hospital Medical Center. The system continuously analyzed EHR information for current ED patients and recommended potential candidates for clinical trials. Relevant patient eligibility information was presented in real time on a dashboard available to CRCs to facilitate their recruitment. To assess the system’s effectiveness, we performed a multidimensional, prospective evaluation for a 12-month period, including a time-and-motion study, quantitative assessments of enrollment, and postevaluation usability surveys collected from the CRCs.

**Results:**

Compared with manual screening, the use of ACTES reduced the patient screening time by 34% (*P*<.001). The saved time was redirected to other activities such as study-related administrative tasks (*P*=.03) and work-related conversations (*P*=.006) that streamlined teamwork among the CRCs. The quantitative assessments showed that automated screening improved the numbers of subjects screened, approached, and enrolled by 14.7%, 11.1%, and 11.1%, respectively, suggesting the potential of ACTES in streamlining recruitment workflow. Finally, the ACTES achieved a system usability scale of 80.0 in the postevaluation surveys, suggesting that it was a good computerized solution.

**Conclusions:**

By leveraging NLP and machine learning technologies, the ACTES demonstrated good capacity for improving efficiency of patient identification. The quantitative assessments demonstrated the potential of ACTES in streamlining recruitment workflow and improving patient enrollment. The postevaluation surveys suggested that the system was a good computerized solution with satisfactory usability.

## Introduction

### Background

Clinical trials are experiments in biomedical research involving human subjects. These trials advance medical science and are a valuable step toward providing new treatments. According to ClinicalTrials.gov, there are 34,240 clinical trials actively recruiting subjects in the United States [1]. However, challenges with patient recruitment for clinical trials are recognized as major barriers to the timely and efficacious conduct of translational research [2-8]. In current practice, clinical trial staff (eg, clinical research coordinators; CRCs) manually screen patients for eligibility before approaching them for enrollment. The process includes reviewing the patients’ electronic health records (EHRs) for demographics and clinical conditions, collating and matching the information to trial requirements, and identifying eligible candidates based on the requirements [3]. One critical hurdle is the lack of an efficient method for detecting subjects who meet eligibility criteria [2,5,8]. Given the large volume of data documented in EHRs, it is labor-intensive for the staff to screen relevant information, particularly within the time frame needed. For patients presenting during clinical visits, screening would ideally take place early enough in the visits such that the eligible candidates could be approached for enrollment without prolonging their stay. The workflow not only poses a significant financial burden for an institution undertaking clinical research, but also hinders the successful completion of clinical studies if eligible candidates cannot be approached [9].

In recent years, automated patient screening for clinical trials has become an active area for research and development and several informatics-based approaches have been proposed. These approaches either (1) manually design rule-based triggers for a clinical trial (eg, International Classification of Diseases-9 codes) to identify patient cohorts [10-14] or (2) automatically match patterns (eg, symptoms and diseases) between clinical trial description and EHR information to identify potential trial-patient matches [15-22]. Rule-based triggers are widely used in current practice in the form of trial-specific best practice advisories, but their accuracy remains an issue [23]. Automated matching methods rely on advanced technologies such as natural language processing (NLP) to improve the accuracy of subject identification [15-22]. However, these applications are usually experimental and their performance in clinical practice remains unclear [24]. Few studies explicitly report patient screening efficiency in prospective settings. Consequently, even though manual screening is inefficient, it is currently a standard practice in conducting clinical trial research.

In our recent work, we developed an NLP- and machine learning–based system, Automated Clinical Trial Eligibility Screener (ACTES), to automate subject identification for clinical trials [18,19,25]. The system extracted patient demographics and clinical assessments (eg, diagnostic tests) from structured EHR data. It also identified patients’ clinical conditions and treatments (eg, symptoms, diseases, and surgery history) from unstructured clinical narratives using NLP and machine learning technologies. Leveraging information retrieval algorithms, the system matched the extracted content with the eligibility criteria to determine patients’ suitability for clinical trials. The ACTES addressed the problem that is cognitively challenging for humans because of the large volume of data that must be reviewed in a short time. In a gold standard-based retrospective evaluation of 13 pediatric trials, the system achieved statistically significant improvement in screening efficiency and suggested a potential reduction in staff workload [18]. It was further validated on a set of 55 pediatric oncology trials, where a similar reduction in screening effort was observed [19]. To test its generalizability on external data sources, the ACTES was submitted to the 2018 National NLP Clinical Challenges (Track 1) that aimed to automate identification of adult patients for 13 clinical trial criteria (eg, myocardial infarction and advanced cardiovascular disease) [26]. The ACTES achieved an overall performance of 90.3% (micro F-measure) that was placed in a statistical tie with the top 5 out of 101 systems [27]. Although the system achieved promising results in patient identification, the imperfection of NLP technologies in understanding language semantics (eg, word sense disambiguation) and syntax (eg, assertion detection) caused multiple types of false positive recommendations [18,19]. Additional study is therefore required to investigate their impact on system integration and end user satisfaction.

To this end, we integrated the ACTES into the institutional workflow to support real-time patient screening. To evaluate its effectiveness on patient recruitment, we implemented a multidimensional evaluation, including a time-and-motion study, quantitative assessments of enrollment, and postevaluation usability surveys. A time-and-motion study is a continuous, observational study where an observer watches the subject (eg, a CRC) performing a task and uses a timekeeping device to record the time taken to accomplish the task [28]. The methodology has been used to evaluate the efficiency of clinical activities to reduce redundant work and improve workflow [29-31]. Results of time-and-motion analysis can also identify positive and negative effects of new technologies during their workflow integration [32-34]. The postevaluation surveys were implemented with system usability scale (SUS), which is a standardized questionnaire measuring the users’ perceived usability on computerized solutions [35]. The SUS is a widely used and validated survey instrument and it has been applied to assess the usability of patient-oriented computerized programs in prior clinical studies [36-38].

### Objective

This study sought to evaluate the ACTES’s impact on the institutional workflow, prospectively and comprehensively. We hypothesized that compared with the manual screening process, using EHR-based automated screening would improve efficiency of patient identification, streamline patient recruitment workflow, and increase enrollment in clinical trials. Specific aims of this study were (1) to evaluate the effects of ACTES on improving patient screening via an observational, randomized time-and-motion study, (2) to assess the system’s impact on patient recruitment using quantitative assessments of enrollment, and (3) to identify the system’s advantages and limitations with postevaluation usability surveys. This study is among the first to investigate real-time integration of the NLP- and machine learning-based patient screening into clinical practice. Our long-term objective is to develop an automated system that will contribute to a more efficient and scalable paradigm in clinical trial enrollment across health care institutions with an EHR in place.

## Methods

### Setting and Participants

The pediatric emergency department (ED) at Cincinnati Children’s Hospital Medical Center (CCHMC) is an urban, level 1 trauma center with more than 70,000 patient visits annually. The department is an appropriate place for many clinical studies because of the variety and complexity of presenting complaints and varied patient demographics [39]. The ED staffs 8 full-time CRCs (including a CRC manager) to recruit subjects for clinical studies from 8 am to midnight, 6 days a week, and from 8 am to 5 pm on Sundays. Owing to the unplanned nature of ED visits, CRCs have to manually screen and enroll patients during each visit, without an opportunity to preplan or sort. The average length of stay in the CCHMC ED is 3.4 hours. Given the fluctuating patient volumes in this busy clinical environment, although ample potential research subjects are presented, there is little time for the CRCs to repetitively review EHRs, locate clinical staff to answer questions regarding patients’ conditions or treatments, and approach eligible candidates for enrollment. For these reasons, in the study, we focused on the integration of ACTES into the ED. The EHR in use during the study period was the Epic Systems.

The ethics approval for this study was provided by the CCHMC institutional review board (study ID: 2013-4241). After system integration, we performed a prospective study between October 1, 2017 and September 30, 2018, which involved a total of 46,612 patient visits during CRC staffing time. A total of 7 CRCs consented to and participated in the study by using the ACTES during their workday and providing feedback. As the CRC manager supervised the staff and had little involvement in patient screening, he was excluded from our study.

### Clinical Trials

During the study period, there were 6 clinical trials actively recruiting patients in the CCHMC ED. The trials required review of either structured data (eg, demographics, vital signs, medications, and procedure orders) or patients’ clinical conditions from unstructured narrative notes (eg, chief complaints, signs, and symptoms) or both for enrollment. The clinical trials covered a variety of diseases, including respiratory tract infection, traumatic brain injury, and serious bacterial infections. The summary of these clinical trials and their core eligibility criteria are presented in Multimedia Appendix 1.

### Patient Recruitment With Automated Screening

**Figure 1:**
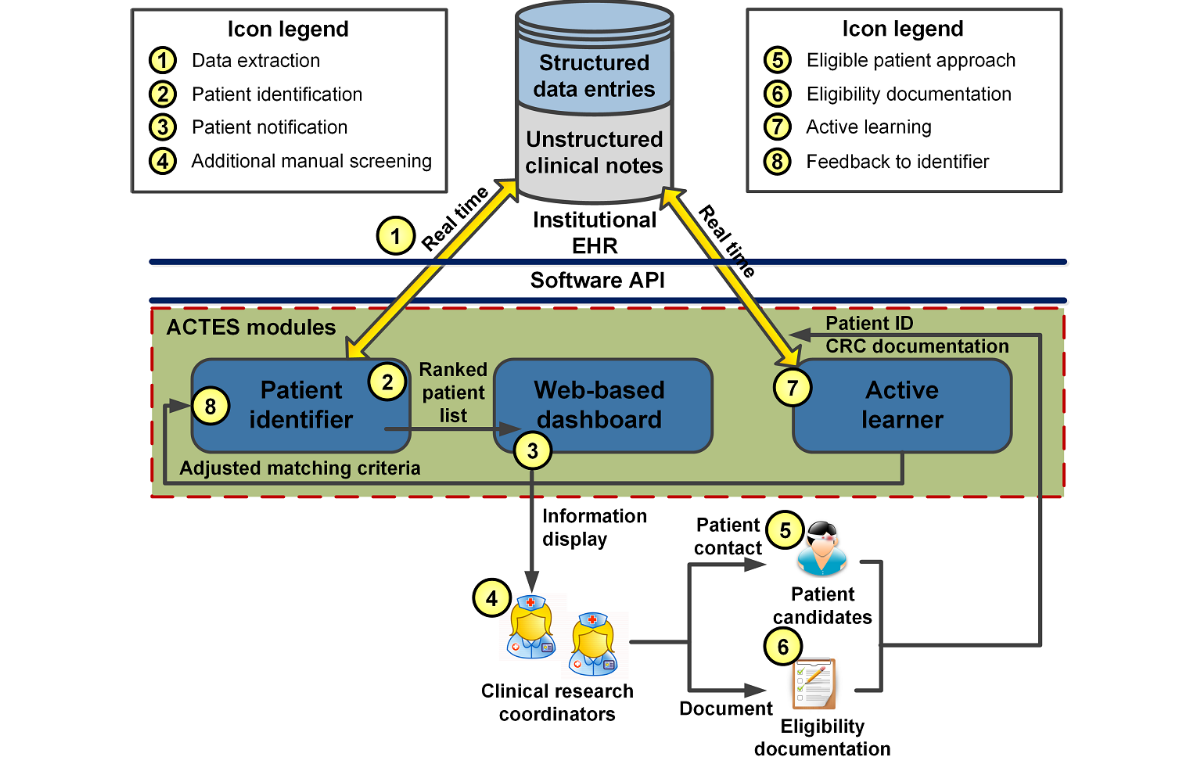
The overview of patient recruitment workflow with automated patient screening. API- Application Programming Interface; ACTES: Automated Clinical Trial Eligibility Screener; CRC: Clinical Research Coordinator; EHR: Electronic Health Record.

We leveraged a human factors engineering framework to design the recruitment workflow with automated patient screening [40]. The process involved an iterative design of system modules with the CRC team using a series of group meetings. Figure 1 diagrams an overview of the patient recruitment workflow, where the ACTES modules are highlighted in blue. Details of the module functionalities can be found in our earlier publications [18,19,27].

Patient information was recorded routinely in the EHR as structured entries (eg, vital signs) and unstructured clinical notes (eg, signs, symptoms) as per standard clinical workflow. We did not modify either the content or the structure of how the clinical entries were created. The ACTES ran continuously on a secured, Health Insurance Portability and Accountability Act-compliant server to extract structured and unstructured entries from the EHR for current ED patients (process 1). Given the EHR information, the system first excluded patients whose structured entries did not meet trial inclusion requirements. The structured entries included age, sex, race, language, legal guardian presence, vital signs, acuity, medication, and procedure orders (Multimedia Appendix 1). The complete sets of codes (eg, Current Procedural Terminology codes) for medication and procedure orders were provided by the clinical trial investigators. For the remaining patients, the system identified relevant information (eg, symptoms) from unstructured clinical narratives using NLP technologies. Details of the NLP process have been specified in our earlier studies [18,19]. To summarize, the clinical narratives were first tokenized and lemmatized, where duplicate sentences and punctuations were removed. The system then identified relevant phrases (eg, symptom-related keywords) from the text and extracted their medical concepts from clinical terminologies, including concept unique identifiers from the Universal Medical Language System, Systematized Nomenclature of Medicine—Clinical Terms codes, and a standardized nomenclature for clinical drugs [41-43]. Assertion (negation, temporal, and experiencer) detection was applied to convert the extracted terms to the corresponding format. For example, the phrase *to rule out pneumonia* was converted to *NEG_C0032285* in assertion detection. The same process was applied to identify phrases and medical concepts from unstructured trial requirements. Finally, information retrieval algorithms matched between the extracted terms and ranked patient candidates based on the degree of matching (process 2). The ranked list of patients along with their demographics and clinical information were displayed on a Web-based dashboard available to the CRCs (process 3). The information was refreshed at 10-min increments to accommodate real-time updates. Given the recommended patients as potential subjects for a clinical trial, the CRCs performed additional EHR screening to confirm the candidates’ eligibility before enrollment (process 4). If an eligible candidate was identified, the CRC would document the patient’s eligibility and approach him or her for enrollment before discharge (processes 5 and 6). If a patient was deemed to be not eligible, the CRC would briefly document the reason. The CRC documentation was fed to the active learner in real time (process 7). The module used active learning technologies to analyze the documentation and patient EHRs to find pertinent information associated with eligibility [18]. For instance, the active learner extracted an informative term *skull fractures* (concept unique ID: c0037304) automatically from the EHR of an eligible patient for the clinical trial *M-TBI* (Multimedia Appendix 1) to supplement the definition of *head injury* in the inclusion. This information was leveraged to adjust the trial criteria, which were used to match future candidates during patient identification (process 8).

### Prospective Evaluations

To assess the system’s impact on the CRC workflow, we performed a multidimensional, prospective evaluation that included a time-and-motion study, quantitative assessments of enrollment, and postevaluation usability surveys collected from the CRCs.

#### The Time-and-Motion Study

To evaluate the system effects on improving patient screening efficiency, we performed an observation-based, randomized time-and-motion study in the ED. One observer tracked how a CRC allocated his or her time during a 120-min observation section at 30-second increments. In each section, the observer shadowed the CRC to observe the patient recruitment workflow. Overall, 1 or 2 major activities that the CRC was engaged in were recorded in each 30-second period. At the end of the section, the observer calculated the percentage of time the CRC spent on each activity.

The list of activities performed by the CRCs was developed in our earlier study [9]. The major activities included patient screening, patient contact, performing procedures, waiting, and other activities, each of which has multiple subcategories. A research assistant independent of the CRC team was hired as the observer to avoid potential biases in activity documentation. The observer shadowed the CRCs step by step without conversation to mitigate the Hawthorne effect [44].

The study included 96 observation sections distributed evenly among CRCs and staff shifts within 4 1-month periods. Each 1-month period comprised 24 observation sections, where the ACTES was used to facilitate patient screening on 12 sections stratified sampled based on the CRCs and staff shifts. The 4 time periods covered the fall (October 2017), winter (February 2018), spring (April 2018), and summer (August-September 2018) to mitigate seasonal effects on patient recruitment. We compared the percentage of time spent on CRC activities (eg, patient screening) with and without using the ACTES. The statistical significance of the difference in time spent per activity was assessed using unpaired *t* test [45].

#### Quantitative Assessments of Enrollment

In the ED, potentially eligible candidates could be missed momently if the CRCs were busy screening and enrolling other subjects. We hypothesized that by improving efficiency of patient identification, the ACTES would subsequently improve patient recruitment. To this end, we calculated 3 enrollment statistics as follows: (1) patients screened, as defined by the number of patients for whom the CRCs reviewed a significant portion of the EHR (eg, demographics, chief complaints, and procedure orders), (2) patients approached, as defined by the number of patients physically approached by the CRCs for enrollment, and (3) patients enrolled, as defined by the number of patients enrolled for a trial. The statistics were aggregated on a weekly basis. The enrollment statistics were then compared with historical controls documented in the CRC study database that was routinely used to record screening and enrollment information. For each clinical trial, the enrollment statistics when using ACTES were compared with that of the same time period in the previous year when the ACTES was not in place. The results were assessed individually and in aggregate; unpaired *t* tests were used to evaluate the statistical significance of the difference in enrollment performance.

#### Postevaluation Usability Surveys

Usability is the effectiveness, efficiency, and satisfaction with which users can perform a specific set of tasks in a particular environment [46]. It is one of the most important factors that impact users’ adoption and meaningful use of health information technologies [47]. As our ultimate goal is to disseminate the ACTES across health care institutions, we evaluated the system usability periodically in the study to inform its future refinement.

After each 1-month time-and-motion evaluation, the CRCs were asked to complete a postevaluation usability survey, including the SUS and a set of open-ended questions. The templated usability survey is presented in Multimedia Appendix 2. The SUS comprised 10 statements on a 5-point agreement scale between *strongly disagree* and *strongly agree* [35]. On the basis of earlier research, a score of 68 is considered to be average with higher scores reflecting greater than average usability across comparable applications. The SUS results were analyzed quantitatively to assess the usability of ACTES over time. The open-ended questions were analyzed qualitatively to identify advantages and limitations of the ACTES and to refine the system design and user interface.

## Results

### Time-and-Motion Study

**Figure 2:**
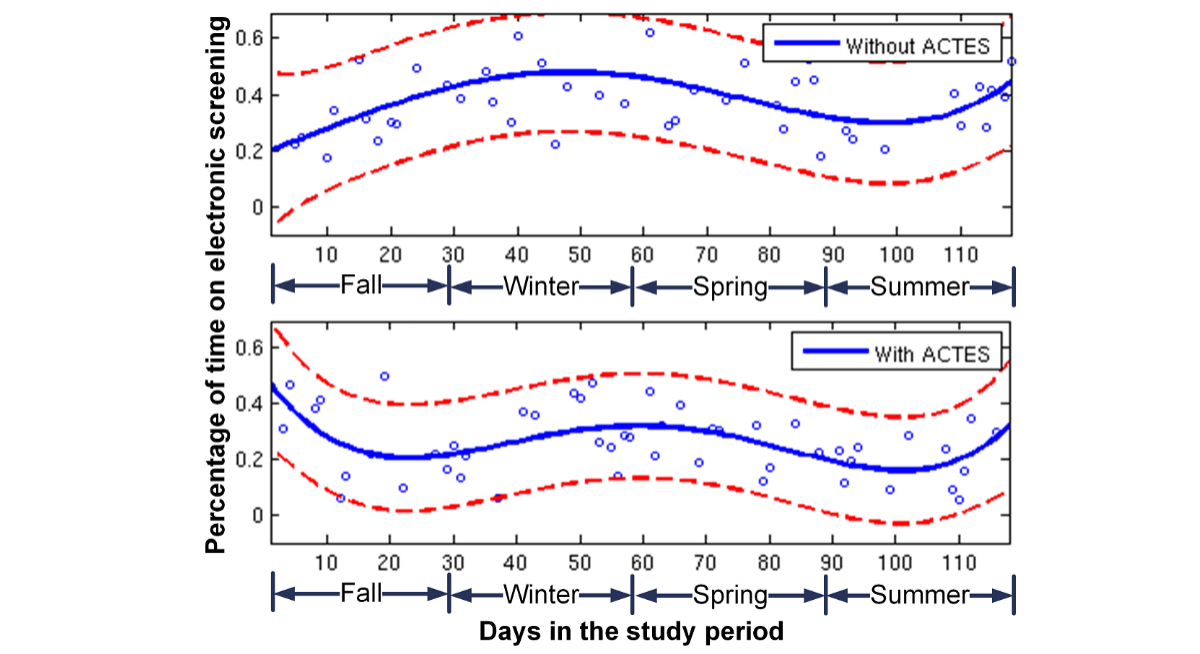
The percentage of time on electronic screening along study days. ACTES: Automated Clinical Trial Eligibility Screener.

Table 1 presents the percentage of time spent on CRC activities averaged over all observation sections. The CRCs spent 38.5% of time on electronic screening without the ACTES. The time was reduced statistically significantly to 25.6% when the ACTES was in place (*P*<.001). Figure 2 illustrates a regression analysis on time for electronic screening along the study days. Without using the ACTES, the screening time increased in the winter and decreased in the spring and summer. With using the system, the screening time decreased gradually, with a mild increase in the winter season.

**Table 1:** Percentage of time spent on clinical research coordinator activities with and without using automated patient screening.

Category and clinical research coordinator activities		With ACTES^a^, %	Without ACTES, %
**Patient screening**			
	Electronic screening (browsing electronic health record or ACTES)	25.6^b^	38.5
	In-person screening (with physician, nurse, and patient)	1.5	2.1
	Logging patient eligibility in study databases	5.2	6.6
	Nonelectronic screening (reviewing log sheet)	0.2^b^	0.4
**Patient contact**			
	Introducing study	0.5	0.4
	Consent procedures	0.9	0.4
	Unclassified patient contact	0.0	0.3
**Performing study procedures**			
	Clinical research coordinator performing study procedures and collecting data (eg, interviews, sample collection)	5.9	5.3
**Waiting**			
	Waiting for clinical procedures to be completed	0.6	0.5
	Waiting for sample collection to be completed	1.5^b^	0.5
	Other unspecified waiting	1.2	0.8
**Other activities**			
	Study-related admin tasks (eg, reviewing study packet, preparing supplies)	15.8^b^	10.9
	Work-related conversations	10.5^b^	6.6
	Miscellaneous work-related admin tasks	4.7	4.6
	Emails/Web browsing	11.1	8.8
	Walking	7.1	6.3
	Personal time (nonwork-related activities)	7.6	6.9

^a^ACTES: Automated Clinical Trial Eligibility Screener.

^b^The difference between clinical research coordinator activities in a category is statistically significant at the .05 level.

**Table 2:** The average numbers of subjects screened, approached, and enrolled per week with and without automated patient screening.

Trial abbreviation	With automated screening			Without automated screening		
	Screened	Approached	Enrolled	Screened	Approached	Enrolled
Biosignature	29.4^a^	2.0	1.2	25.3	2.0	1.4
CARPE-DIEM	62.6^a^	6.9	4.2	54.5	8.2	5.2
ED-STARS	17.5	8.8	6.7	17.2	7.8	5.8
HealthyFamily	52.4^a^	39.0^a^	4.3	44.1	33.8	4.1
M-TBI	10.1	0.9	0.8	12.3^b^	1.3	0.5
Torsion	4.0^a^	1.1	2.4^a^	2.2	0.9	1.5
Average	29.6	10.1	3.0	25.8	9.1	2.7

^a^The enrollment statistics with automated screening is significantly higher than that without automation (*P*<.05).

^b^The enrollment statistics with automated screening is significantly lower than that without automation (*P*<.05).

In addition to electronic screening, the overall patient screening time by CRCs was reduced from 47.6% without ACTES to 32.5% with ACTES (*P*<.001). The saved time was redirected to work-related activities, including waiting for sample collection (*P*=.03), study-related administrative tasks (*P*=.03), and work-related conversations (*P*=.006).

### Quantitative Assessments of Enrollment

Table 2 shows the average numbers of subjects screened, approached, and enrolled per week with and without the automated patient screening. Compared with historical controls, using the ACTES resulted in more screened patients averaged over all trials (*P*=.08). The improvements were statistically significant for the majority of clinical trials. The use of ACTES also improved the numbers of approached and enrolled patients, although the difference was statistically significant for only a couple of clinical trials (*HealthyFamily* and *Torsion*).

### Postevaluation Usability Surveys

Table 3 presents the SUS scores averaged over the CRCs after each time-and-motion evaluation. The total SUS score was 67.9 when ACTES was first in place, suggesting it to be an acceptable computerized application [35]. By the end of the study period, the score was improved to 80.0, which represented a good computerized solution. The ratings to individual SUS statements reflected different aspects of the system’s usability and the CRCs’ satisfaction in using the application.

**Table 3:** The average scores of system usability scale given by the clinical research coordinator participants.

Statements	Five-point scale (1-5)^a^, mean (SD)			
	Fall^b^	Winter^c^	Spring^d^	Summer^e^
1. I would like to use this system frequently.	2.4 (1.1)	3.2 (1.1)	3.7 (0.9)	3.2 (0.6)
2. I found the system unnecessarily complex.	2.1 (1.0)	1.8 (1.4)	1.5 (0.5)	1.4 (0.7)
3. I thought the system was easy to use.	4.6 (0.5)	4.5 (0.5)	4.7 (0.5)	4.7 (0.5)
4. I would need the support of a technician to use this system.	1.7 (1.1)	1.1 (0.4)	1.2 (0.4)	1.1 (0.4)
5. The various functions in the system were well integrated.	3.3 (0.6)	3.3 (1.1)	3.8 (0.4)	3.7 (0.7)
6. I thought there was too much inconsistency in this system.	3.1 (1.1)	3.6 (1.0)	3.3 (0.7)	2.1 (1.0)
7. Most people would learn to use this system very quickly.	4.5 (0.8)	4.5 (0.5)	4.5 (0.5)	4.4 (0.8)
8. I found the system very cumbersome to use.	3.3 (1.3)	2.3 (0.9)	2.0 (1.0)	1.9 (0.9)
9. I felt very confident using the system.	4.0 (1.3)	4.2 (0.5)	4.7 (0.5)	4.0 (1.2)
10. I needed to learn a lot of things before I could use this system.	1.3 (0.4)	1.6 (0.5)	2.2 (1.3)	1.4 (0.4)

^a^1 indicates *strongly disagree* and 5 *strongly agree*.

^b^Overall score of system usability scale (SUS): 67.9.

^c^Overall score of SUS: 72.5.

^d^Overall score of SUS: 78.0.

^e^Overall score of SUS: 80.0.

## Discussion

### Principal Findings

Compared with traditional manual screening, using the ACTES significantly reduced the screening time by 34% (Table 1). The saved time was redirected to other activities such as administrative tasks and work-related conversations that streamlined teamwork among the CRCs. The regression analysis on the screening time illustrated the known seasonal effects on patient recruitment. Owing to an increase in patient volume during viral respiratory seasons (the fall and winter), the time increased without the ACTES, which was expected from prior time trends in the ED. In comparison, the time decreased gradually with the ACTES, reflecting the CRCs’ learning curve on adopting new technologies. Projecting the regression results to future data, we estimated to have a 50% reduction in screening effort when the CRCs fully adopt our system. These promising observations suggested continued benefits gained from automated patient screening. In addition, ACTES will enable the development of a continual, 24-h screening service, which could facilitate recruitment of subjects during nonstaffing periods (including approximately one-third of patient visits).

Compared with historical controls, the enrollment statistics with ACTES further confirmed its effectiveness on improving CRC screening efficiency (Table 2). We observed that automated screening was more useful for clinical trials with multiple conditions (eg, *HealthyFamily*) and vague eligibility description (eg, *CARPE-DIEM* and *Torsion*). In a busy clinical environment, it was difficult for the CRCs to memorize a variety of clinical conditions and match them to a large volume of patients. Use of ACTES could ease eligibility memorization and improve the screening efficiency, particularly in these complex studies. However, the system could be less helpful for the trials that included only demographics criteria (eg, *ED-STARS*) and for those that required chart reviewing of EHR information that is not available to the system (eg, imaging results required by *M-TBI*). In addition to improvement in screening efficiency, the system also showed potential to streamline recruitment workflow by improving patient approach and enrollment.

The postevaluation surveys demonstrated the usability of ACTES on several fronts. The system was easy to learn (Statement 3 in Table 3), easy to use (Statement 7), and its functions were well-integrated (Statement 5). All CRCs felt confident in using the system (Statement 9). In particular, the CRCs’ satisfaction in using the system improved over time, once they adapted to this new technology (Statement 1).

### Areas of Improvement

Systematic error analyses have been performed on retrospective data in our previous research to identify limitations of the ACTES [18,19]. In this study, we focused on identifying areas of improvement based on the CRC feedback. The SUS suggested that there was inconsistency in system recommendation (Statement 6 in Table 3). This is because of the false positive recommendations made by the NLP technologies (eg, miss of negation detection), which has been identified as a limitation in our retrospective studies. To alleviate this problem, we have developed additional regular expressions for assertion detection and used *bag-of-phrases* matching technologies to balance sensitivity and specificity [27]. Advanced NLP algorithms will be explored in future iterations to improve the detection of semantic and temporal relations within the context.

In addition, the system was rated slightly cumbersome to use (Statement 8) when it was first in place. By analyzing feedback in the postevaluation surveys, we observed that it was because of the lack of functionalities on the dashboard (eg, a function for hiding clinical trials not actively enrolling on a day). Additional functions were implemented thereafter to meet the CRC needs.

Finally, the majority of CRCs indicated an information delay on patient recommendation (Question 3 in the open-ended questionnaire; Multimedia Appendix 2). We hypothesize that this is because of the lag in documentation by health care providers early in a patient visit, where the progress notes were not delivered to the ACTES in a timely fashion. For instance, a patient might be recommended for *HealthyFamily* hours after he or she had been triaged for asthma (an inclusion criterion). This could be because the physician filed the patient’s progress note after he or she was admitted, in which case the CRCs were never able to approach that candidate for enrollment. As shown in the literature, delayed documentation is a frequent finding in a high acuity and busy clinical environment [48,49]. As ACTES relies on data entered by EHR users, any strategies that facilitate timely clinical documentation will improve the system usability as well. Coordinating the clinical workflow to accelerate information delivery both for patient care and our system warrants further investigation.

Although the ACTES significantly improved patient screening efficiency, the problems described above occasionally delayed the CRCs’ decision making and negatively affected their satisfaction. Consequently, the users’ attitudes toward using the system remained slightly better than neutral (Statement 1 in Table 3). To improve the CRCs’ willingness of system use, the suggested areas of improvement have been adopted to inform our next development phase.

### Limitations of the Study

One limitation of the study is that it included only 6 clinical trials running in a single clinical department. Although the included trials covered a variety of diseases, they generally did not contain complex logics (eg, criteria involving analysis of laboratory results). To assess its generalizability, we plan to integrate the system into other units (eg, oncology department) in our institution that conduct more complicated clinical studies. In addition, although the study demonstrated the benefits gained from automated patient screening, it did not assess the cost of system implementation because of the intermittent development cycle. In the future, we will perform appropriate cost-benefit analyses when implementing the system in other clinical units and health care institutions. Limited by the study length, the statistical power on quantitative assessments was not sufficiently high. To address this limitation, we will continue collecting enrollment statistics from the ED to generate power to detect significant differences. Finally, project planning and communication are in progress to evaluate the ACTES on a more diversified patient population (eg, adult patients), in multiple institutions, and with clinical data under different formats (eg, data from different vendor EHRs).

### Conclusions

We designed and integrated an NLP- and a machine learning–based system, ACTES, into the ED and prospectively studied its impact on patient recruitment. In an observation-based, randomized time-and-motion study, the system demonstrated good capacity for improving efficiency of patient identification. The quantitative assessments demonstrated the potential of ACTES in streamlining recruitment workflow and improving patient enrollment. The postevaluation surveys suggested that the system was a good computerized solution with satisfactory usability. The promising results from our multidimensional evaluation confirmed the effectiveness of automated patient screening in prospective clinical settings. As such, we hypothesize that the ACTES, when rolled out for dissemination, will provide significant benefits to nationwide research networks and health care institutions in executing clinical research by harnessing the EHR data in real time.

## Appendix

Multimedia Appendix 1 The clinical trial descriptions and their core eligibility criteria.

Multimedia Appendix 2 The templated postevaluation usability survey.

## Abbreviations

ACTES: Automated Clinical Trial Eligibility Screener
CCHMC: Cincinnati Children’s Hospital Medical Center
CRC: clinical research coordinator
ED: emergency department
EHR: electronic health record
NLP: natural language processing
SUS: system usability scale
