# Supplementary material for: A Real-Time Automated Patient Screening System for Clinical Trials Eligibility in an Emergency Department: Design and Evaluation
Source: JMIR Med Inform. 2019 Jul 24;7(3):e14185. doi: 10.2196/14185 (PMC6685132; doi:10.2196/14185)
Supplement: Multimedia Appendix 1 [file medinform_v7i3e14185_app1.pdf]

Appendix 1. The clinical trial descriptions and their core eligibility criteria.

Title	Abbreviation	Objective	Core inclusion criteria	Core exclusion criteria
RNA biosignatures: a paradigm change for the management of young febrile infants	Biosignature	To determine if new laboratory methods looking for biological markers in the blood could be a faster and more accurate way to determine if a child has a viral infection or bacterial infection	0-60 days old (S[a]); rectal temperature of 100.4°F or higher within past 24 hours (S); patient is being evaluated for serious viral/bacterial infections with blood screening tests including blood culture (U[b])	Premature birth (S); received antibiotics within 4 days of emergency department presentation (U); overwhelming clinical sepsis (U); presence of a major congenital anomaly (U); presence of inborn errors of metabolism (U); presence of a congenital heart disease (U); presence of chronic lung disease (U); presence of a disease that would affect the immune system (U); presence of indwelling catheters/shunts (U)
Catalyzing Ambulatory Research on Pneumonia Etiology and Diagnostic Innovations in Emergency	CARPE-DIEM	To catalyze ambulatory research on pneumonia etiology and diagnostic innovations in emergency medicine	Aged between 3 months and 18 years (S); legal guardian should be present (S); chest x-ray ordered to rule out pneumonia (S); signs and symptoms of a lower respiratory tract infection (U); evidence of	Immunodeficiency or immunosuppression (U); chronic pulmonary disease (U); chronic cardiac disease (U); sickle cell disease (U); neuromuscular disorders (U); history of aspiration pneumonia (U)

Medicine			acute infection (U)	
Emergency Department Screen for Teens At Risk for Suicide	ED-STARS	To develop an optimal suicide risk screen for adolescents presenting to the emergency department	Aged between 12 and 17 years (S); legal guardian should be present (S); patient has one or more of the following symptoms (case group): mental disorders, psychiatric disorders, behavioral disorders, depression, and suicidal ideation (U); patient does not have the above symptoms (control group; U)	Mentally too unstable (U); patient has cognitive impairment (U); patient has migraine or headache (U)
An intervention to reduce second hand smoke exposure among pediatric	HealthyFamily	Caregivers who smoke and their children who present to the emergency department with a possible secondhand smoke exposure-related illness	Aged between 0 and 17 years (S); overall acuity 2-5 (S); legal guardian should be present (S); patient has one or more the following symptoms: allergic rhinitis, apparent life-threatening event, asthma, bronchitis,	Patient has a previous tracheostomy (U)

emergency patients			bronchiolitis, cold (flu) symptoms, chest congestion, cough, sore throat, tonsillitis, croup, stridor, difficulty in breathing, nasal congestion, sinusitis, ear drainage, ear pain, ear infection, pink eye, eye irritation, upper respiratory infection, and wheezing (U)	
Investigating diagnostic and prognostic Markers of early Traumatic Brain Injury	M-TBI	To demonstrate the ability of specific acute serum biomarkers to distinguish children with traumatic brain injury and associated injury on head CT[c] from children with orthopedic injury	Aged between 14 days and 4 years (S); legal guardian should be present (S); witnessed or concerns for abusive head trauma with signs and symptoms of head injury (U); head CT ordered (S); abnormality on head CT (eg, intracranial injury or fracture; U)	Penetrating traumatic brain injury (U); history of previous brain injury or encephalopathy (U); conditions that may predispose to fracture or bleeding (U)

Scoring system in the acute scrotum	Torsion	To validate a clinical scoring system for diagnosing testicular torsion	Aged between 9 and 18 years (S); English-speaking (S); legal guardian should be present (S); patient has acute scrotal pain for less than 3 days (U)	Patient has a previous testicular surgery (U)

[a]S: A criterion on structured electronic health record data fields.

[b]U: A criterion on unstructured electronic health record data fields.

[c]CT: Computed tomography.
