# Supplementary material for: A Real-Time Automated Patient Screening System for Clinical Trials Eligibility in an Emergency Department: Design and Evaluation
Source: JMIR Med Inform. 2019 Jul 24;7(3):e14185. doi: 10.2196/14185 (PMC6685132; doi:10.2196/14185)
Supplement: Multimedia Appendix 2 [file medinform_v7i3e14185_app2.pdf]

Appendix 2. The templated post-evaluation usability survey.

1) ACTES Usability Questionnaire

Please user the ACTES service [https://xxxxxxxxxxxxxxxxxxxxxxxxxxx] and provide feedback for the following tasks:

1. Is the information organized by the ACTES system useful for identifying/excluding patients?

- ☐ Yes
- ☐ No
- ☐ Other comments

Comments: ____

2. Questions about ACTES recommendations

2.1 Were the "hit terms" and "exclusion terms" useful for you?

- ☐ Yes
- ☐ No
- ☐ Somehow

2.2 Please identify/describe examples of inconsistency in ACTES recommendations when you used the system.

2.3 Did you use patient EHR for additional information? If so, what information you looked at?

2.4 What additional functions/information you would like to see in the ACTES?

3. What are the aspects you DON'T like about the ACTES system?

- ☐ Appearance
- ☐ Information delay
- ☐ Low quality of recommendation (e.g., too many irrelevant hit terms)
- ☐ Lack of sufficient functions
- ☐ Other

Comments: ____________________________________

4. Any additional comments, questions, concerns, suggestions?

2) System Usability Scale

When answering the following questions, please think of using the ACTES system in the PED.

Question	Strongly Disagree				Strongly Agree
1. I think that I would like to use this system frequently.					
2. I found the system unnecessarily complex.					
3. I thought the system was easy to use.					
4. I think that I would need the support of a technical person to be able to use this system.					
5. I found the various functions in this system were well integrated.					
6. I thought there was too much inconsistency in this system.					
7. I would imagine that most people would learn to use this system very quickly.					
8. I found the system very cumbersome to use.					
9. I felt very confident using the system.					
10. I needed to learn a lot of things before I could get going with this system.					
